# Acute Kidney Injury and Post-Artesunate Delayed Haemolysis in the Course of *Plasmodium falciparum* Malaria

**DOI:** 10.3390/pathogens13100851

**Published:** 2024-09-30

**Authors:** Alicja Kubanek, Małgorzata Sulima, Aleksandra Szydłowska, Katarzyna Sikorska, Marcin Renke

**Affiliations:** 1 Division of Occupational, Metabolic and Internal Diseases, University Centre of Maritime and Tropical Medicine, Medical University of Gdansk, 81-519 Gdynia, Poland; alicja.kubanek@gumed.edu.pl (A.K.); aleksandra.szydl@gmail.com (A.S.); mrenke@gumed.edu.pl (M.R.); 2 Division of Tropical and Parasitic Diseases, University Centre of Maritime and Tropical Medicine, Medical University of Gdansk, 81-519 Gdynia, Poland; katarzyna.sikorska@gumed.edu.pl; 3 Division of Tropical Medicine and Epidemiology, Institute of Maritime and Tropical Medicine, Faculty of Health Sciences, Medical University of Gdansk, 81-519 Gdynia, Poland

**Keywords:** malaria, acute kidney injury, haemolysis

## Abstract

Malaria is a major international public health problem. The risk of acquiring malaria varies depending on the intensity of transmission and adherence to mosquito precautions and prophylaxis recommendations. Severe malaria can cause significant multiorgan dysfunction, including **acute kidney injury (AKI).** Intravenous artesunate is the treatment of choice for severe malaria in non-endemic areas. One of the possible events connected with the lifesaving effects of artemisins is post-artesunate haemolysis (PADH), which may be potentially dangerous and under-recognised. We present a case of a seafarer with severe *Plasmodium falciparum* malaria complicated with AKI and PADH, with a good response to steroid treatment. This case highlights the need for malaria prophylaxis in business travellers, e.g., seafarers to malara-endemic regions, and close supervision of patients with malaria even after the completion of antimalarial treatment due to the possibility of late complications.

## 1. Introduction

Malaria is a life-threatening disease spread to humans by *Anopheles* mosquitoes. Globally, in 2022, there were an estimated 249 million malaria cases and 608,000 malaria deaths in 85 countries [1]. According to the guidelines set by the organisation Kidney Disease: Improving Global Outcomes (KDIGO), acute kidney injury (AKI) is defined by changes in kidney function, based on serum creatinine (SCr) increase and/or a decrease in urine output [2,3]. Using different classifications, AKI occurs in around forty percent of adult patients with severe malaria [4] and is even more common in the paediatric population [5]. Malaria can cause disease in glomeruli, tubules, and in the interstitial region [6]. The pathophysiology of AKI in severe malaria is not fully defined. Multiple pathological processes such as parasite sequestration, endothelial dysfunction, haemolysis-mediated oxidative stress, and immune-mediated damage contribute to this condition [7,8]. Intravenous artesunate is the treatment of choice for severe malaria in non-endemic areas, with high efficacy and reasonable safety reported [9,10,11]. One of the possible events connected with the lifesaving effects of artemisins is post-artesunate haemolysis (PADH), which may be potentially dangerous and under-recognised [12,13,14]. The aim of this study is to present the difficulties associated with the treatment of malaria, including the occurrence of late complications.

## 2. Case Presentation

A 60-year-old seafarer with a previous history of hypertension presented with high fever, nausea, and diarrhoea. The symptoms had lasted for four days prior to hospital admission and had occurred after a business stay in Angola. Due to the COVID-19 pandemic, there was an extended transfer home, and he had spent two days in a hotel in Luanda. He received all the recommended pre-travel vaccinations but did not take malaria prophylaxis. There was no previous history of malaria infections, kidney or liver disease, transfusions, or auto-immune disease. He was receiving no other medications apart from hypotensive agents. Ten days after returning home, a fever up to 38 °C, gastrointestinal symptoms, and malaise occurred. He had been treated with amoxicillin/clavulanic acid with no improvement.

## 3. Investigations

**Figure 1:**
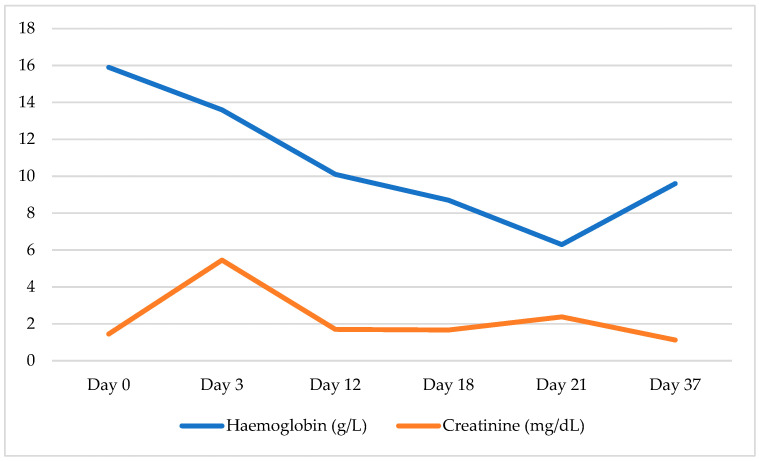
Haemoglobin and creatinine levels in the course of infection.

On initial presentation, the patient appeared to have deterioration of auto- and allopsychic orientation, a Glasgow coma scale (GCS) of 14/15, mild dehydration and visceral icterus, blood pressure of 140/80 mmHg, heart rate of 90 beats per minute, and oxygen saturation of 98%. An immuno-chromatographic test detecting parasite lactate dehydrogenase of Plasmodium species OptiMAL-IT (DiaMed, Cressier, Switzerland) was positive. Subsequent tests including blood smears and real-time polymerase chain reaction (RT-PCR) confirmed a diagnosis of severe *Plasmodium falciparum* malaria, based on hyperparasitaemia (10% of erythrocytes infected in blood films). The haemoglobin (Hb) level was 15.9 g/dL, serum creatinine (sCr) was 1.45 mg/dL, eGFR was 49 mL/min (MDRD formula), and CRP was 175 mg/L; a urinalysis revealed proteinuria, haemogobinuria, and the presence of bilirubin. HIV and hepatitis C antibodies and hepatitis B surface antigen were negative. The laboratory findings are shown in Table 1. Haemoglobin and creatinine levels in the course of infection are presented in Figure 1. Abdominal ultrasound scans revealed liver enlargement, the kidneys were of normal size, and echogenicity and cortical medullary differentiation were within the normal range.

On admission, treatment with intravenous artesunate (2.4 mg/kg body weight) and oral doxycycline was started and continued for three days. Since the coexisting bacterial infection could not be excluded at the moment, intravenous ceftriaxone was added to the therapy; parenteral fluids and heparin were also provided. Repeated blood and urine cultures were negative. During this time, diarrhoea occurred, and a *Clostridium difficile* toxin test was positive. The antibiotic therapy was modified, cephalosporin was discontinued, and oral vancomycin was administered. On day 3, increases in bilirubin concentration (8.13 mg/dL) and sCr (5.46 mg/dL) were observed; at this time, the haemoglobin level was 13.6 g/dL. Oral antimalarial treatment was continued (total dose of 400 mg of artemether and 2400 mg of lumefantrine—AL). Subsequent blood smears for malaria were negative. Twelve days after the initiation of intravenosus treatment with artesunate, the patient developed moderate anaemia (Hb 10.1–8.9 g/dL) with biochemical indicators of haemolysis, elevated reticulocytes (31.3‰), lactate dehydrogenase (LDH) 1420 U/L, and plasma cell-free haemoglobin (449 mg/L). In a urine analysis, no abnormalities were observed at that time. The patient was discharged, recovering well; the Hb level was stable, and the platelet count was within the normal range (217 G/L), with downtrending bilirubin (8.13–2.81 mg/dL) and inflammation markers, as well as decreasing sCr (1.5 mg/dL). A close outpatient follow-up was recommended, including a control blood count, kidney function parameters, and haemolysis indicators, to be undertaken within one week.

The patient was readmitted to the hospital after 5 days due to worsening anaemia and weakness. He reported the occurrence of dark urine in the days prior to hospitalisation (18 days after artesunate treatment); diuresis was preserved, and the hydration status was normal. A physical examination revealed skin/conjunctival pallor, jaundice, and papular rash on the trunk and upper limbs. Laboratory findings were consistent with haemolytic anaemia (Hb 6.3 g/dL, reticulocytes 65.3‰, schistocytes present on the peripherial blood smear, bilirubin 2.8 mg/dL, LDH 1958 U/L, haptoglobin < 0.1 g/L, cell-free haemoglobin 696 mg/L). During this time, the sCr was 2.3 mg/dL; a urinalysis showed haemoglobinuria, proteinuria (1.9 g/L), leukocyturia, and the presence of granular casts; and urinary osmolarity was 326 mOsm/kgH_2_O. The biochemical parameters of inflammation were slightly increased (CRP 25 mg/L, procalcitonin 1.1 ng/mL); blood and urine cultures, as well as repeated blood smears for *Plasmodium falciparum*, were negative. Abdominal ultrasounds showed normal kidney echogenicity and cortical medullary differentiation and a prostate gland volume of 30 mL, with post-void residual volume less than 50 mL. Serum complement levels were within the normal range, platelet count was preserved, and disintegrin and metalloprotease with thrombospondin 1 repeats (ADAMTS13) activity was not decreased. Glucose-6-phosphate dehydrogenase (G6PD) was qualitively normal. Due to the suspicion of a non-immunological mechanism of PADH, the patient received two units of packed red blood cells. After transfusions, deterioration of the general condition, increase in haemolysis parameters, and no increment in haemoglobin levels was observed. A direct antiglobulin test (DAT) was positive for complement, and cold agglutinin was detected in plasma. Due to the lack of improvement after the previous treatment and the suspicion of acute tubulointerstitial nephritis, steroid therapy was initiated (total dose of 1.5 g methyloprednisone intravenously, followed by oral form with 0.5 mg/kg in reduced doses). The patient received adequate fluids, a proper hydration status was maintained, and urine output was preserved; we also administered acetaminophen. Biochemistry revealed downtrending creatinine and urea concentrations, no hyperkalaemia, and compensated metabolic acidosis. Renal replacement therapy was not required. In the following days, the clinical condition improved, and compensatory polyuria was observed, with a normal electrolytes range and decreasing haemolysis parameters. On discharge, the Hb level was 9.6 g/dL, sCr was 1.1 mg/dL, eGFR > 60 mL/min/1.73 m^2^, and CRP < 1.0 mg/L. During further outpatient follow-up, normalisation of haemoglobin levels and kidney parameters was observed. The patient’s condition was good, and he returned to work as a sailor.

## 4. Discussion

The clinical manifestations of severe malaria may be various, and its assessment is crucial to obtain optimal treatment results [9,10]. The pathomechanism of haemolytic anaemia following malaria is multifactorial, and determining its cause may be challenging. This report described a case of severe malaria followed by post-artesunate delayed haemolysis and AKI. Taking into consideration the number of malaria cases, AKI remains an important clinical issue in the course of the infection [4,5]. Since the pathomechanism of haemolysis and AKI in malaria is complex, its proper treatment remains difficult. In the presented case, at the time of diagnosis, mild haemolysis was observed. The patient was diagnosed with severe malaria due to confusion, hyperparasitaemia, and AKI. At that moment, the most probable mechanism of AKI was both pre-renal, due to dehydration, and renal, induced by haemolysis, causing acute tubular injury [6]. As a result of the treatment, kidney function improved, and the Hb levels remained stable. At readmission, 18 days after artesunate administration, symptoms of severe haemolysis and AKI were observed. A diagnosis of postartesunate delayed haemolysis (PADH), which could cause kidney injury via a non-immunological mechanism, was most probable. Moreover, the occurrence of a rash, haemoglobinuria, leukocyturia, and non-nephrotic proteinuria and the presence of granular casts were consistent with the suspicion of immune-mediated tubulointerstitial nephritis caused by the medication. Acute tubular injury (ATI) secondary to ischemia and cell-free haemoglobin direct action were taken into consideration too. Pre-renal injury was unlikely because no signs of dehydration were present, and the urinary osmolarity was <350 mOsm/kgH_2_O. The platelet count and complement levels were normal, as well as ADAMTS13 activity, which did not indicate the diagnosis of haemolytic uremic syndrome or trombotic thrombocytopenic purpura.

AKI caused by haemolisis after artesunate treatment is a rare complication [13,14]. PADH is usually defined as the presence of haemolysis with a >10% decrease in haemoglobin level or a >10% rise in LDH concentrations occurring more than 8 days after the initiation of treatment [15]. Artesunate reduces parasitaemia by targeting the ring-stage malaria parasite inside the erythrocyte. PADH is probably caused by haemolysis of damaged erythrocytes (known as pitted erythrocytes) after the removal of the nonvital parasite by the spleen. It can lead to haemolytic events 1 up to 4 weeks after the implementation of anti-malarial treatment and is generally self-limiting. Some patients require transfusions. Additionally, drug-induced autoimmune mechanisms of PADH, caused by the use of parenteral AS, is taken into account [16,17]. DAT positivity due to malaria itself can also be detected as the result of a nonspecific expression of malaria-related systemic immune activation [18]. In the reported case, there was a possibility of drug-induced autoimmune mechanisms due to the use of ceftriaxone as well. Such cases in patients with falciparum malaria have been reported [19,20]. In our patient, other causes of haemolytic anaemia, including glucose-6-phosphate dehydrogenase deficiency, were ruled out. Coombs’ positive haemolytic anaemia test after AS is probably underreported. In a study presenting a series of 36 patients treated with parenteral artesunate, PADH occurred in more than a quarter of cases (27.8%) [16]. In another group, the incidence was 27%, and, despite the high prevalence, anaemia remained mild in 85% of cases [17]. Particular attention should be paid to performing DAT when haemolysis after artesunate administration is detected. PADH is more frequent in nonimmune returning travellers presenting with high parasitaemia [15]. What needs to be emphasised is that PADH may complicate the course of uncomplicated *P. falciparum* malaria in patients treated with oral artemisin-derivative drugs [21,22]. The legitimacy of the use of steroids in PADH has not been evaluated, although reports of steroids used in cases of autoimmune haemolytic anaemia associated with malaria infection have been published [21,23,24]. Patients with malaria have significantly increased lipid peroxidation. Artesunate exerts an anti-parasitic effect by increasing oxidative stress in the parasite. While increasing oxidative stress is an effective mechanism of action for parasite elimination, it can cause extensive damage to host cells and tissues, probably contributing to pathologic complications. In some studies, it was shown that acetaminophen decreased oxidant injury in the kidneys, improved renal function, and reduced renal damage in rhabdomyolysis-induced renal failure [25,26]. In the presented case, the indication for steroid use was the suspicion of interstitial nephritis in the presence of positive DAT. A kidney biopsy was not performed; however, the good response to the steroid treatment indicates the involvement of the above-mentioned background.

Since the long-term adverse outcomes after kidney injury may be serious, proper follow-up of patients is required [27]. Taking into account a possible occurrence of PADH as a complication of malaria treatment, it is worth considering that haemoglobin levels should be assessed weekly for 1 month after artesunate administration.

## 5. Conclusions

A close follow-up of patients after malaria treatment is obligatory due to the possibility of late complications. This case study shows that a patient with severe malaria and suspected interstitial nephritis and PADH, in the presence of positive DAT, had a good response to steroid use. This case highlights the need for malaria prophylaxis in business travellers, e.g., seafarers traveling to malara-endemic regions.

## Figures and Tables

**Table 1:** Laboratory results. Day 0 is defined as the first day of patient observation.

Parameter	Day 0	Day 3	Day 12	Day 18	Day 21	Day 37	Normal Range
Haemoglobin (g/L)	15.9	13.6	10.1	8.7	6.3	9.6	13.0–18.0
Hematocrit (%)	46.4	38.6	28	25.8	18.5	27.8	40–52
Platelets (×10^3^/μL)	29	53	335	196	228	217	150–400
Cell-free haemoglobin (mg/L)	-	-	449	-	696	-	<20 in plasma <50 in serum
Plasma haptoglobin (g/L)	-	-	-		<0.1	-	0.3–2
Creatinine (mg/dL)	1.45	5.46	1.7	1.67	2.38	1.13	0.7–1.3
Urine (mg/dL)	67	161	48	43	-	-	18–55
Lactate dehydrogenase (U/L)	647	689	-	1420	1958	425	125–220
Total bilirubin (mg/dL)	4.74	8.13	2.14	2.81	2.81	0.9	0.3–1.2
Potassium (μmol/L)	3.7	3.4	5.2	4.4	4.4	3.9	3.5–5.1
CRP (mg/L)	175.7	239.3	15	42.1	25.8	<1.0	<5.0
Procalcitonin (ng/mL)	10.94	26.96	-	-	-		<0.5
Venous pH	7.412	7.428	-	-	7.386	-	7.35–7.45
Venous bicarbonate (mmol/L)	15	20.8	-	-	19.9	-	22–28

## Data Availability

The original contributions presented in the study are included in the article, further inquiries can be directed to the corresponding authors.

## References

[1] WHO (2023). World Malaria Report 2023.

[2] Kidney Disease: Improving Global Outcomes (KDIGO) (2012). Acute Kidney Injury Work Group KDIGO Clinical Practice Guideline for Acute Kidney Injury. Kidney Int. Suppl..

[3] Lameire N.H., Levin A., Kellum J.A., Cheung M., Jadoul M., Winkelmayer W.C., Stevens P.E., Conference Participants (2021). Harmonizing acute and chronic kidney disease definition and classification: Report of a Kidney Disease: Improving Global Outcomes (KDIGO) Consensus Conference. Kidney Int..

[4] Marks M., Armstrong M., Walker D., Doherty T. (2014). Imported falciparum malaria among adults requiring intensive care: Analysis of the literature. Malar. J..

[5] Batte A., Berrens Z., Murphy K., Mufumba I., Sarangam M.L., Hawkes M.T., Conroy A.L. (2021). Malaria-Associated Acute Kidney Injury in African Children: Prevalence, Pathophysiology, Impact, and Management Challenges. Int. J. Nephrol. Renovasc. Dis..

[6] Silva Junior G.B.D., Pinto J.R., Barros E.J.G., Farias G.M.N., Daher E.F. (2017). Kidney involvement in malaria: An update. Rev. Inst. Med. Trop. Sao Paulo.

[7] Katsoulis O., Georgiadou A., Cunnington A.J. (2021). Immunopathology of Acute Kidney Injury in Severe Malaria. Front. Immunol..

[8] Hawkes M.T., Leligdowicz A., Batte A., Situma G., Zhong K., Namasopo S., Opoka R.O., Kain K.C., Conroy A.L. (2022). Pathophysiology of Acute Kidney Injury in Malaria and Non-Malarial Febrile Illness: A Prospective Cohort Study. Pathogens.

[9] WHO (2010). Guidelines for the Treatment of Malaria.

[10] WHO (2015). Guidelines for the Treatment of Malaria.

[11] Roussel C., Caumes E., Thellier M., Ndour P.A., Buffet P.A., Jauréguiberry S. (2017). Artesunate to treat severe malaria in travellers: Review of efficacy and safety and practical implications. J. Travel Med..

[12] Rolling T., Agbenyega T., Krishna S., Kremsner P.G., Cramer J.P. (2015). Delayed haemolysis after artesunate treatment of severe malaria—Review of the literature and perspective. Travel Med. Infect. Dis..

[13] Leong K.W., Singh K.P., Leder K., Tong S.Y.C. (2021). Acute kidney injury secondary to severe delayed haemolysis in intravenous artesunate use for severe malaria. BMJ Case Rep..

[14] Plewes K., Haider M.S., Kingston H.W., Yeo T.W., Ghose A., Hossain M.A., Dondorp A.M., Turner G.D., Anstey N.M. (2015). Severe falciparum malaria treated with artesunate complicated by delayed onset haemolysis and acute kidney injury. Malar J..

[15] Jauréguiberry S., Ndour P.A., Roussel C., Ader F., Safeukui I., Nguyen M., Biligui S., Ciceron L., Mouri O., Kendjo E. (2014). Postartesunate delayed haemolysis is a predictable event related to the lifesaving effect of artemisinins. Blood.

[16] Camprubí D., Pereira A., Rodriguez-Valero N., Almuedo A., Varo R., Casals-Pascual C., Bassat Q., Malvy D., Muñoz J. (2019). Positive direct antiglobulin test in post-artesunate delayed haemolysis: More than a coincidence?. Malar. J..

[17] Jauréguiberry S., Thellier M., Ndour P.A., Ader F., Roussel C., Sonneville R., Mayaux J., Matheron S., Angoulvant A., Wyplosz B. (2015). Delayed-onset hemolytic anaemia in patients with travel-associated severe malaria treated with artesunate, France, 2011–2013. Emerg. Infect. Dis..

[18] Ascoli Bartoli T., Lepore L., D’Abramo A., Adamo G., Corpolongo A., Scorzolini L., Giancola M.L., Bevilacqua N., Palazzolo C., Mariano A. (2021). Systematic analysis of direct antiglobulin test results in post-artesunate delayed haemolysis. Malar. J..

[19] Plewes K., Maude R.J., Ghose A., Dondorp A.M. (2015). Severe falciparum malaria complicated by prolonged haemolysis and rhinomaxillary mucormycosis after parasite clearance: A case report. BMC Infect. Dis..

[20] Savargaonkar D., Das M.K., Verma A., Mitra J.K., Yadav C.P., Srivastava B., Anvikar A.R., Valecha N. (2020). Delayed haemolysis after treatment with intravenous artesunate in patients with severe malaria in India. Malar. J..

[21] De Nardo P., Oliva A., Giancola M.L., Ghirga P., Mencarini P., Bibas M., Nicastri E., Antinori A., Corpolongo A. (2013). Haemolytic anaemia after oral artemether-lumefantrine treatment in a patient affected by severe imported falciparum malaria. Infection.

[22] Tsuchido Y., Nakamura-Uchiyama F., Toyoda K., Iwagami M., Tochitani K., Shinohara K., Hishiya N., Ogawa T., Uno K., Kasahara K. (2017). Development of Delayed Hemolytic Anaemia After Treatment with Oral Artemether-Lumefantrine in Two Patients with Severe Falciparum Malaria. Am. J. Trop. Med. Hyg..

[23] Lebrun D., Floch T., Brunet A., Julien G., Romaru J., N’Guyen Y., Cousson J., Giltat A., Toubas D., Bani-Sadr F. (2018). Severe post-artesunate delayed onset anaemia responding to corticotherapy: A case report. J. Travel Med..

[24] Raffray L., Receveur M.C., Beguet M., Lauroua P., Pistone T., Malvy D. (2014). Severe delayed autoimmune haemolytic anaemia following artesunate administration in severe malaria: A case report. Malar. J..

[25] Boutaud O., Moore K.P., Reeder B.J., Harry D., Howie A.J., Wang S., Carney C.K., Masterson T.S., Amin T., Wright D.W. (2010). Acetaminophen inhibits hemoprotein-catalyzed lipid peroxidation and attenuates rhabdomyolysis-induced renal failure. Proc. Natl. Acad. Sci. USA.

[26] Janz D.R., Bastarache J.A., Peterson J.F., Sills G., Wickersham N., May A.K., Roberts L.J., Ware L.B. (2013). Association between cell-free haemoglobin, acetaminophen, and mortality in patients with sepsis: An observational study. Crit. Care Med..

[27] See E.J., Jayasinghe K., Glassford N., Bailey M., Johnson D.W., Polkinghorne K.R., Toussaint N.D., Bellomo R. (2019). Long-term risk of adverse outcomes after acute kidney injury: A systematic review and meta-analysis of cohort studies using consensus definitions of exposure. Kidney Int..

